# Renal function monitoring in heart failure – what is the optimal frequency? A narrative review

**DOI:** 10.1111/bcp.13434

**Published:** 2017-10-22

**Authors:** Ahmed Al‐Naher, David Wright, Mark Alexander John Devonald, Munir Pirmohamed

**Affiliations:** ^1^ The Wolfson Centre for Personalised Medicine The University of Liverpool Liverpool UK; ^2^ Institute of Cardiovascular Medicine and Science Liverpool Heart and Chest Hospital Liverpool UK; ^3^ Renal and Transplant Unit Nottingham University Hospitals NHS Trust Nottingham UK

**Keywords:** clinical decision systems, diuretics, drug safety, heart failure, kidney failure, renal function monitoring

## Abstract

The second most common cause of hospitalization due to adverse drug reactions in the UK is renal dysfunction due to diuretics, particularly in patients with heart failure, where diuretic therapy is a mainstay of treatment regimens. Therefore, the optimal frequency for monitoring renal function in these patients is an important consideration for preventing renal failure and hospitalization. This review looks at the current evidence for optimal monitoring practices of renal function in patients with heart failure according to national and international guidelines on the management of heart failure (AHA/NICE/ESC/SIGN). Current guidance of renal function monitoring is in large part based on expert opinion, with a lack of clinical studies that have specifically evaluated the optimal frequency of renal function monitoring in patients with heart failure. Furthermore, there is variability between guidelines, and recommendations are typically nonspecific. Safer prescribing of diuretics in combination with other antiheart failure treatments requires better evidence for frequency of renal function monitoring. We suggest developing more personalized monitoring rather than from the current medication‐based guidance. Such flexible clinical guidelines could be implemented using intelligent clinical decision support systems. Personalized renal function monitoring would be more effective in preventing renal decline, rather than reacting to it.

## What is Already Known about this Subject

- Diuretics can lead to acute kidney injury, which is one of the most common causes of hospital admission due to adverse drug reactions.
- There have been no published studies investigating the optimal frequency of renal function monitoring in patients with heart failure on diuretics and other potential nephrotoxic agents.

## What this Study Adds

- There is variability in national and international guidelines regarding monitoring of renal function in heart failure patients with many of these being based on expert opinion.
- Personalized guidance schemes for monitoring renal function based on patient and disease characteristics, including drug treatment, need to be developed.

## Introduction

Heart failure is an important cause of morbidity and mortality in the UK, and is likely to increase in prevalence as the population ages. Heart failure incidence overall is 1%, rising to around 12% in patients over 75 years. It currently affects up to 900 000 patients in the UK 1. These patients often suffer from comorbidities including diabetes, chronic obstructive pulmonary disease and ischaemic heart disease resulting in frequent hospital admissions and greater burden on the health service 2. One of the most common causes of hospital admission and deterioration in these patients is worsening renal function 3. Prevalence of chronic kidney disease (CKD) ranges from 39 to 60% in heart failure cohorts and is associated with increased mortality and morbidity 4, 5. Renal function can be overlooked due to lack of consensus on optimal timing and frequency of monitoring. National Institute for Health and Care Excellence (NICE) guidelines for management of chronic heart failure recommend a general rule of 6‐monthly blood tests for urea and electrolytes in stable patients. However renal deterioration can occur rapidly and unpredictably and it is likely that monitoring is highly variable. Clearer guidance for monitoring renal function may be helpful in reducing admissions by facilitating earlier intervention such as modification of drugs and their doses which may be contributing to the renal decline. Development of such guidelines is needed in this particular patient group because many drugs used in the treatment of heart failure contribute to renal decline 6.

Here we review reasons for renal decline in heart failure syndromes and explore the evidence for renal function monitoring, including optimal frequency.

## Relationship between heart failure and renal failure

An association between decline in cardiac function and renal function is well documented. Heart failure itself is associated with high risk of renal dysfunction and development of CKD 7, 8, 9. Conversely, poor renal function has been shown to predict left ventricle (LV) dysfunction 10. This means that the onset of CKD is a risk factor for the subsequent development of heart failure 11, 12. There is even evidence to suggest that estimated glomerular filtration rate (eGFR) correlates strongly with LV function well before any diagnosis of renal failure or heart failure has been made 13, 14, 15. Indeed, up to a quarter of patients with CKD have symptoms suggestive of heart failure before a formal diagnosis is made 16. Collectively, the bidirectional link between cardiac and renal function can lead to clinical presentations that are termed cardio‐renal syndrome (CRS).

There are five types of CRS, the classification of which reflects the presumed primary and secondary problem 17:

- **Type 1**: Acute heart failure causes acute kidney injury (AKI)
- **Type 2**: Chronic heart failure causes CKD
- **Type 3**: AKI or acute renal failure causes acute cardiac failure
- **Type 4**: CKD causes chronic cardiac dysfunction, including heart failure
- **Type 5**: An acute or chronic systemic disorder causes both cardiac and renal failure (e.g. sepsis, diabetes mellitus, systemic lupus erythematosus)

The mechanism of renal injury in CRS is not clear but is likely to be multifactorial. The interaction between heart and renal failure can be described in terms of heart failure pathophysiology, renal responsive changes and the drugs to which patients are exposed.

### Heart failure pathophysiology causing renal decline

Heart failure states cause increased central venous pressure (CVP). As the ventricles dilate and cardiac output decreases, preload increases, which is reflected in increased CVP. This venous back pressure can be transmitted to the renal vasculature causing chronic renal venous congestion, which in turn reduces glomerular blood flow by reducing the pressure gradient between afferent and efferent arterioles 18. The observed association between high CVP states and fall in renal function is consistent with this paradigm 19.

In addition, right ventricular dilation in heart failure (which increases CVP) lowers cardiac output by several mechanisms. First, the direct effect of over‐dilation causes decreased ventricular contractility (Frank–Starling relationship) 20. Secondly, dilation of the right ventricle impairs LV filling by increasing LV extramural pressure, reducing LV functional volume and reducing preload (the reverse Bernheim phenomenon 21). The resulting reduction in cardiac output decreases renal perfusion and measured renal function.

### Structural and responsive renal changes during heart failure

Changes in renal perfusion are likely to account for much of the renal deterioration in patients with heart failure. However, the situation can be aggravated by drugs used to manage heart failure such as diuretics, which reduce intravascular volume and renal perfusion 22. Compensatory mechanisms are activated to attempt to maintain intravascular pressure and preserve renal perfusion. Chronic compensation puts stress on normal regulatory systems, increasing risk of progressive failure. An important example is the renin–angiotensin–aldosterone (RAA) system, which is activated by low renal arteriolar pressure, causing secretion of angiotensin, which promotes vasoconstriction, and aldosterone, which promotes sodium retention 23. Long‐term activation of the RAA system is harmful, consistent with the reduced mortality associated with angiotensin‐converting enzyme inhibitor (ACEi) use in patients with heart failure and their renoprotective effect in some types of nephropathy. The mechanism by which chronic RAA system activation causes harm is not clear, but contributing factors are likely to include systemic vasoconstriction, which increases cardiac afterload, further reducing the cardiac output of a dilated heart. This leads to an even greater reduction in renal perfusion, causing further activation of the RAA system and a vicious cycle of deterioration 24.

### Medications used in heart failure

Drugs used to manage heart failure can reduce renal function by various mechanisms. This is particularly relevant in CRS types 1 and 2 where heart failure leads to renal failure.

#### Loop diuretics

These drugs promote natriuresis by reducing sodium reabsorption via the NKCC transporter in the loop of Henle, resulting in an efflux of water and reduction of intravascular volume. While this is beneficial for symptomatic heart failure with volume overload, it decreases total blood volume and thus reduces blood pressure contributing to renal hypoperfusion and compensatory systemic and renal vasoconstriction to maintain blood pressure. Since the function and perfusion of glomeruli rely on a constant flow from the renal arterioles, any reduction in flow in this way leads to renal decline, and if the patient's compensatory mechanisms fail, it can quickly deteriorate into AKI 25.

#### Thiazide diuretics

These drugs inhibit the sodium–chloride transporter in the distal tubule. Their hypotensive effects may also be useful for achieving blood pressure targets in patients with ischaemic heart disease while the naturietic effect can have protective effects against pulmonary oedema. The hypokalaemic effect can counter‐balance the potassium raising effects of ACEis/angiotensin receptor blockers (ARBs) and potassium‐sparing diuretics 26, 27.

As with loop diuretics, renal function can deteriorate acutely as a consequence of the hypovolaemia, resulting in AKI, or gradual progression of CKD. Hypokalaemia results from increased sodium delivery to the cortical collecting duct with consequent increased uptake by the sodium epithelial channel ENaC and an increase in potassium excretion via the channel ROMK2 to maintain electrical neutrality. In addition, the diuretic‐induced naturesis causes upregulation of ENaC, which is aldosterone‐sensitive. Enhanced ENaC activity also increases cortical collecting tubule acid secretion, which can cause metabolic alkalosis, which itself can be aggravated sometimes in renal failure because of impaired acid–base homeostasis 28, 29.

#### ACEis and ARBs

Many studies have shown a prognostic benefit of ACEis/ARBs in both heart failure and renal failure. Their effect on blocking the RAA system causes a predictable increase in serum creatinine because of their effect on intraglomerular haemodynamics. As serum creatinine currently remains the main biomarker for measuring renal filtration function, an acute decline in renal function might be apparent on initiation or dose escalation of these drugs. The extent of the rise in serum creatinine may indicate whether it is a physiological or pathological response, with a total rise of up to 20% generally considered acceptable. Combination of ACEis/ARBs with other *high risk* medications and a background of pre‐existing renal disease may cause subsequent renal deterioration, if not titrated appropriately 30, 31.

#### Beta‐blockers

Bisoprolol is the most commonly used β‐blocker in heart failure, and is known to improve prognosis 32, probably through a combination of both sympathetic inactivation, resulting in downregulation of the RAA system, as well as reduction in endothelin‐1 and thromboxane prostaglandins, which promote vasoconstriction in response to sclerosis and injury. These effects result in renal arteriole vasodilation, which improves blood flow and protects renal perfusion 33. Renal decline, however, can occur with β‐blockers due to a reduction in cardiac output, consequent to the bradycardia, which could reduce renal perfusion 34, 35. However, bisoprolol has largely been accepted as being safe over a long‐term period in renal failure 36, 37. Indeed, it continues to be of prognostic benefit even in renal decline, and this benefit is even greater in patients with the most severe stages of renal failure, without affecting overall eGFR significantly 38.

#### Calcium channel blockers

Heart failure patients often suffer from hypertension, especially if the primary cause is due to ischaemic heart disease. These patients are likely to be prescribed hypotensive agents such as calcium channel blockers. By reducing systemic blood pressure, these agents may also carry a potential risk of reducing perfusion and filtration pressure through the kidney, causing renal ischaemia and decline in function over time 39. However, in practice, amlodipine seems to have renoprotective effects in CKD patients, especially when paired with ARBs 40, 41, probably due to a reduction in renal artery smooth muscle contraction leading to a higher renal flow, even while systemic blood pressure is reduced 42. Indeed, even a single dose of amlodipine can lead to a demonstrable increase in eGFR in CKD patients 43.

#### Aspirin

This is commonly used in secondary prevention of ischaemic heart disease because of its antiplatelet effects. Rarely, aspirin can cause an idiosyncratic reaction causing tubulo‐interstitial nephritis, which can lead to AKI. This is rare at low doses of 75 mg, although the risk is slightly higher if combined with other nonsteroidal anti‐inflammatory drugs (NSAIDs) and analgesics 44. Similar to other NSAIDs, aspirin at high doses can be nephrotoxic because of detrimental effects on renal prostaglandins. It can also cause fluid retention, which can exacerbate heart failure 45.

#### Aldosterone antagonists

Spironolactone and eplerenone have shown significant benefit in heart failure outcomes, but they can also lead to serious adverse effects. As with loop and thiazide diuretics, they can increase risk of dehydration and hypoperfusion. They also cause potassium retention which can lead to hyperkalaemia, the risk being higher in CKD. Hyperkalaemia increases risk of arrhythmias, morbidity and mortality. Concurrent use of aldosterone antagonists with ACEis increases risk of hyperkalaemia and so should be used with caution 7.

#### Digoxin

This is now used infrequently in patients with heart failure, and is used mainly in patients with concomitant atrial fibrillation. Digoxin has positive inotropic and negative chronotropic effects. There have been very few studies of the effects of digoxin on renal function in patients with heart failure and CKD, but it seems to have no effect on renal dysfunction in small doses. Conversely, since digoxin excretion is mainly renal, accumulation can occur in severe kidney dysfunction, leading to digoxin toxicity and potentially cardiac arrhythmias 29.

#### Hydralazine and nitrates

These drugs both increase nitric oxide (NO) availability in blood. NO is a potent vasodilator of systemic vasculature which lowers blood pressure and potentially increases renal arterial flow. This effect has been demonstrated during intravenous administration in an acute setting 46. ISDN combined with hydralazine has been shown to decrease mortality in patients with renal failure 47. Hydralazine can rarely cause drug‐induced lupus, which can also involve the kidneys leading to renal dysfunction 48. Hydralazine is renally excreted and can accumulate in patients with CKD 49.

#### Ivabradine

This is used to reduce heart rate in patients already taking β blockers, who remain symptomatic. This drug acts on the I_f_ sodium channel in the sinoatrial node to delay repolarization. Only 20% of ivabradine is renally excreted, with no known associations with renal pathology making it unlikely to pose a renal risk 32.

## The biomarker manifestations of renal decompensation

AKI is a rapid deterioration in renal function, usually over days to weeks. It is defined arbitrarily by a rise in serum creatinine or a fall in urine output, according to internationally accepted criteria such as the KDIGO classification. In clinical practice it is far more common to use the serum creatinine criteria, as hourly urine output is rarely recorded routinely outside intensive care and renal units. AKI is first defined as an increase in serum creatinine by 50% from baseline within 7 days, or an absolute increase in creatinine by 26.5 μmol l^–1^ over 48 h, or the presence of acute oliguria (<0.5 ml kg–1 h^–1^ for 6 h) 50. It is then staged (1–3) by further specific creatinine or urine output criteria. AKI can progress to more severe forms with oliguria and need for renal replacement therapy. AKI is a common cause of hospital admission for patients with heart failure. In many cases, the development of AKI, or increase in its severity, may be preventable with appropriate monitoring of renal function and adjustment of prescribed drugs. Earlier detection of AKI by laboratory or clinical parameters allows earlier intervention and probably increased chance of prevention or amelioration.

One early warning system is the clinical classification of worsening renal function (WRF). WRF is a state of pre‐AKI that is specific to heart failure patients. It has a more gradual biomarker (creatinine) increase than AKI, occurring over 6–12 months rather than 7 days, which makes it easier to detect on routine screening than the criteria for AKI 51. WRF is associated with greater mortality, morbidity and hospitalization rate attributable to both renal and heart failure. WRF acts as an early warning system for progression of renal impairment, and allows for earlier intervention so that hospital admission can be avoided 52.

There are various definitions of WRF described in the literature, generally accepted ones being: 25% increase in baseline creatinine; 26.5 μmol l^–1^ absolute increase in creatinine; or 20% decrease in eGFR. While these criteria are similar to AKI definition, the important difference is the much longer timescale. More recently, WRF has been divided into three classes based on severity of renal decline (Table 1) 24 increasing up to an outcome of AKI.

**Table 1:** The accepted biomarker definitions of worsening renal function (WRF) classes compared to Kidney Disease Improving Global Outcomes (KDIGO) Acute Kidney Injury (AKI) definition 50, 60

	WRF Class I	WRF Class II	WRF Class III	AKI
**% increase in creatinine**		25%		50% (over 7 days)
**Creatinine raw value increase (μmol l** ^**–1**^ **)**	17.7–26.5	26.5–44.2	>44.2	>26.5 (over 48 h)
**eGFR raw value decrease (ml min** ^**–1**^ **1.73 m** ^**–2**^ **)**	5–11	11–15	>15	
**% decrease in eGFR**		20%		
**Timescale**	6–12 months	6–12 months	6–12 months	7 days/48 h

The definition of WRF relies on biomarker change from an established baseline 53. Therefore, true risk of renal decline must take into account baseline renal function which is most commonly defined using eGFR formulae 54, 55. This, in turn, influences their risk of further deterioration (Table 2) 56. CKD is graded from G1 (normal) to G5 (end‐stage renal failure): the greater the severity, the greater the risk of mortality from any cause, cardiovascular events and hospitalization 57.

**Table 2:** Grading of CKD definitions based on baseline eGFR (KDIGO CKD definition) 57

GFR category	GFR (ml min^–1^ 1.73 m^–2^)	Renal function
**G1**	>89	Normal or high
**G2**	60–89	Mildly decreased
**G3a**	45–59	Mild – moderate decrease
**G3b**	30–44	Moderate – severe decrease
**G4**	15–29	Severely decreased
**G5**	<15	Kidney failure

As heart failure progresses in patients, it is often accompanied by progressive CKD with a fall in eGFR 9. The patient is at increased risk of WRF and subsequent AKI. This risk is usually compounded by the various drugs and systemic insults to which patients with heart failure are often exposed 7, 58. An effective system of regular monitoring can help clinicians intervene at a stage sufficiently early to reduce risk of progression to kidney failure.

## Frequency of renal monitoring

No study has specifically assessed frequency and optimal timing of renal function monitoring with respect to clinical outcomes in patients with heart failure. Various studies have emphasized the importance of frequent and regular monitoring of renal function but there is no evidence‐based gold standard 59, 60.

To address optimal frequency, published heart failure management guidelines will be reviewed. Other relevant factors will then be taken into account such as renal pharmacodynamics, rate of renal decline, risk factors for renal decompensation and the time required for an intervention to have an effect.

## Methods

The following databases were searched for clinical guidelines for heart failure and studies of renal monitoring in heart failure: Cochrane Database of Systematic Reviews (CSDR), Database of Abstracts of Reviews of Effects (DARE), Cochrane Central Database of Controlled Trials (CENTRAL), MEDLINE, EMBASE, Web of Science and CINAHL. Studies of interest were further reviewed for inclusion of heart failure guidance. Selection of clinical guidelines was agreed by consensus of clinical investigators for relevance to this review. Cited studies within these publications were scrutinized. Search strategy for other related references can be found in the supplementary information (Appendix A).

### Nomenclature of targets and ligands

Key protein targets and ligands in this article are hyperlinked to corresponding entries in http://www.guidetopharmacology.org, the common portal for data from the IUPHAR/BPS Guide to PHARMACOLOGY 61, and are permanently archived in the Concise Guide to PHARMACOLOGY 2015/16 62.

## Renal function monitoring in current guidelines for chronic heart failure

### NICE guidelines for chronic heart failure in adults (Oct 2014) 1

The NICE clinical guidelines for the management of chronic heart failure advise a minimum frequency of 6‐monthly monitoring for patients with stable CHF. Increased frequency (of *days to 2 weeks*) is recommended if changes are made to the drug regimen. More detailed monitoring is suggested for patients with “significant comorbidities or deterioration” though this is not elaborated on further. These are relatively nonspecific monitoring recommendations open to interpretation by clinicians.

For ACEis and ARBs, more specific guidance exists. Checking baseline renal function on initiation of the drug is recommended, with dose titration 2‐weekly, monitoring renal function each time until target dose is reached. Cross‐referencing to NICE clinical guidelines for CKD, renal function monitoring is advised 1–2 weeks after initiation or dose increment. More specific guidance for renal function monitoring in patients with heart failure is given in the appendices (*Appendix D of the NICE guideline for chronic heart failure*). Regular follow‐up monitoring of renal function is advised every 3 months if the patient continues to take ACEis or ARBs. The guidance advises against use of these medications with baseline potassium >5 mmol l^–1^ and recommends discontinuation at ≥6 mmol l^–1^ . Maximum permitted fall in renal function after ACEi/ARB initiation or titration is suggested to be 25% decrease in eGFR or 30% increase in creatinine from pretreatment levels. ACEi or ARB dose should be reduced or the drug discontinued if these limits are exceeded. Any lesser degree of renal deterioration warrants further testing after 1–2 weeks, but the dose should not be decreased unless these limits are exceeded.

The risk of hyperkalaemia with aldosterone antagonists is emphasized with renal function tests suggested postinitiation at 1 week, then at 1, 2, 3, 6 months and then 6‐monthly if stable. Dose should be halved if potassium reaches 5.5–5.9 mmol l^–1^ and stopped if it reaches 6 mmol l^–1^. The guidance also advises frequent monitoring in patients taking loop and thiazide diuretics alongside aldosterone antagonists, as well as advising monitoring of potassium when digoxin is prescribed concurrently, but no frequency is specified.

In summary, the minimum monitoring frequency recommended by NICE is 6‐monthly unless the patient's condition or medication has changed, in which case the minimum interval is reduced to 2 weeks but can be more frequent at the discretion of the clinician. The rationale is that medication for heart failure can cause adverse effects such as dehydration and renal impairment that can manifest within 2 weeks. These recommendations were based on analyses of local data looking at incidence of renal impairment, hospital admission, re‐admission and length of stay in patients where medication had been changed 1.

Evidence used for these suggestions included a study from 1995 in which spironolactone added to ACEis and diuretics resulted in four of 28 patients having a significant rise in creatinine and hyperkalaemia. These changes occurred by first follow up at 4 weeks and were asymptomatic. Therefore, this study recommended weekly monitoring of renal function during initiation and titration of aldosterone antagonists for all patients 63.

Management and monitoring of ACEis, ARBs, β‐blockers and spironolactone in CHF were also addressed by McMurray *et al*. Their recommendations were based on expert opinion and clinical safety trials published before 2001 64. These recommendations were adapted by the NICE Guideline Development Group and further updated in 2010 using evidence from NICE clinical guidance on management of CKD.

### SIGN guideline on management of chronic heart failure (March 2016) 65

The Scottish Intercollegiate Guidelines Network (SIGN) also advises on monitoring of renal function in CHF. For ACEis and ARBs, renal function and electrolyte monitoring is recommended 1–2 weeks after initiation or dose increase. Renal function should then be monitored “frequently and serially until potassium and creatinine have plateaued”, but no specific frequency is suggested.

Maximum acceptable increase in creatinine after ACEis or ARB introduction is considered to be 50% or 266 μmol l^–1^ from baseline with a maximum acceptable serum potassium of 5.5 mmol l^–1^. If these limits are reached, other *high‐risk* agents should be reviewed and discontinued first. If renal function does not improve, ACEis or ARB dose should be halved before re‐checking renal function after 1–2 weeks. If there is still no improvement, specialist opinion should be sought. At no point is there a recommendation to stop ACEis/ARBs and the only contraindications mentioned are angioedema and bilateral renal artery stenosis.

For β‐blockers, renal function monitoring is also recommended 1–2 weeks after initiation or dose change. Dose should be reviewed in the event of hypotension, bradycardia, or fatigue but renal dysfunction is not specifically mentioned.

Guidance regarding aldosterone antagonists is, as with NICE, more comprehensive due to the increased risk of hyperkalaemia. The recommendation is similar to that of NICE with renal function testing at 1 week, then at 2, 3, 4, 6 months then 6‐monthly thereafter if stable. Dose should be halved with serum potassium of 5.5 mmol l^–1^ or above and stopped if above 6 mmol l^–1^. Monitoring with loop diuretics is mentioned but no specific time interval suggested 65.

It is worth noting that this SIGN guidance is also derived from McMurray *et al*.'s practical recommendations for heart failure, the same source used by NICE but follows it more closely than does NICE. This is apparent in the quoted maximum acceptable creatinine rise before intervention is deemed necessary. NICE uses its own limit of 30% increase in creatinine compared with McMurray's (and SIGN's) 50% increase from baseline. It is clear from the wording of the publication by McMurray *et al*. that the higher threshold was designed to prioritize prescription of ACEis, ARBs and cardiovascular outcomes in heart failure rather than discussing renal decompensation 66.

## European Society of Cardiology guidelines for the diagnosis and management of acute and chronic heart failure (May 2016) 67

The European Society of Cardiology (ESC) guidelines acknowledge the high prevalence of CKD in HF and the frequent onset of WRF as a risk factor for future morbidity, AKI and hospital admission. They advise caution when using thiazide and loop diuretics in the context of declining renal function, but they encourage continuation of ACEis and ARBs unless there is a *large decrease* in renal function as a result.

Supplementary information provides some drug‐specific monitoring advice. For ACEis and ARBs, testing is advised 1–2 weeks after initiation and then 1–2 weeks after final titration only (which may be up to five dose titrations later). However, they note that blood chemistry should be monitored “frequently and serially until creatinine and potassium have plateaued”. When stable, regular monitoring is suggested at 4‐monthly intervals. Maximum accepted rise in creatinine is 50% or 266 μmol l^–1^ from baseline. Deterioration above this level should trigger a review of other high‐risk medicines followed by halving of the dose of ACEi or ARB with repeat renal function check after 1–2 weeks. ACEis and ARBs should be discontinued if creatinine increases by 100% or more, or 310 μmol l^–1^ or if eGFR drops below 20 ml min^–1^ 1.73 m^–2^ or if potassium exceeds 5.5 mmol l^–1^.

For aldosterone antagonists, ESC advises renal function and electrolyte checks at baseline, 1 week, then 1, 2, 3, 6, 9 and 12 months after initiation, then 4‐monthly when stable. For hyperkalaemia, they advise halving the dose at 5.5 mmol l^–1^ and discontinuation at 6 mmol l^–1^.

For diuretics, ESC recommends renal monitoring at baseline, then 1–2 weeks after initiation or dose change. Discontinuation of diuretics is recommended in the event of worsening renal impairment or dehydration.

The ESC recommendations are based predominantly on expert opinion. No specific references are given with respect to renal monitoring, although they are similar to NICE and SIGN guidelines (Table 3).

**Table 3:** Summary and comparison of clinical guidelines relating to renal function monitoring in chronic heart failure patients under different circumstances

	NICE	SIGN	ESC	ACCF/AHA
**Stable**	6 monthly	Nonspecific	Nonspecific	Nonspecific
**Clinical deterioration**	Days–2 weeks	1–2 weeks	Nonspecific	Nonspecific
**Change in medications**	Days–2 weeks	1–2 weeks	Nonspecific	Nonspecific
**Digoxin**	Nonspecific	Not mentioned	Nonspecific	Nonspecific
**Aldosterone antagonist**	1 week, then 1, 2, 3, 6 months, then 6 monthly if stable	1 week, then 1, 2, 3, 6 months, then 6 monthly if stable	1 week, then 1, 2, 3, 6, 9, 12 months, then 4 monthly if stable	2–3 days, 7 days, then monthly for 3 months, then 3 monthly if stable
**ACEi/ARBs**	At initiation, 2 weeks, then every 3 months when stable, every dose change	1–2 weeks after initiation or dose change	1–2 weeks after initiation and 1–2 weeks after final dose titration, then 4 monthly when stable	1–2 weeks after initiation or dose change
**Threshold for reviewing ACEi/ARBs or aldosterone antagonist**	Creatinine: >30% increase eGFR: >25% decrease	Creatinine: >50% increase or >266 μmol absolute increase Potassium: >5.5 mmol l^–1^	Creatinine: >50% increase or >266 μmol absolute increase Potassium: >5.5 mmol l^–1^	Development of WRF (Creatinine >25% increase) Potassium: >5.5 mmol l^–1^
**Loop diuretic**	Nonspecific	Nonspecific	At initiation, then 1–2 weeks after initiation and each dose change	Nonspecific
**Thiazide diuretic**	Nonspecific	Nonspecific	At initiation, then 1–2 weeks after initiation and each dose change	Nonspecific
**Hypotensives (β‐blockers)**	Not mentioned	1–2 weeks after initiation or dose change	Not mentioned	Not mentioned
**CKD + ARB/ACEi**	At initiation, 1 week, 2 weeks, dose changes	Nonspecific	Nonspecific	Nonspecific

NICE, National Institute for Health and Care Excellence; SIGN, Scottish Intercollegiate Guidelines Network; ESC; European Society of Cardiology; ACCF/AHA, American College of Cardiology Foundation/American Heart Association; ACEi, angiotensin‐converting enzyme inhibitor; ARB, angiotensin receptor blockers; CKD, chronic kidney disease; WRF, worsening renal function

## ACCF/AHA guideline for the management of heart failure (October 2013) 68

A pragmatic approach to diuretic prescribing is described. It is suggested that furosemide, the most common diuretic in heart failure, is titrated up based on daily weight of the patient to achieve daily weight loss of 0.5–1.0 kg, followed by a lower maintenance dose to keep weight at the target. The dose can then be adjusted in primary care or by the patient, as necessary, over the longer term. For ACEis and ARBs, advice is to start at a low dose then monitor renal function after 1–2 weeks then “periodically thereafter” according to the clinician's judgement, including after dose increases. Risks of renal decline in patients with diabetes and hypertension are noted.

For aldosterone antagonists, a more stringent monitoring regimen is advised, with the option of an alternate day starting dose for patients with CKD and monitoring after 2–3 days then at 7 days for all patients. Thereafter monthly monitoring is advised every 3 months if renal function is stable. This monitoring schedule should then be repeated every time the dose changes.

Sources of this guideline included nine trials involving ACEis, six relating to ARBs, 25 to diuretics and five to aldosterone antagonists. The guideline also cross‐referenced ESC and NICE guidelines.

Compared with other guidelines, advice on renal monitoring is less specific although there is generally a more cautious approach with respect to the risk of worsening renal function. ACEi and ARB guidance is similar to other guidelines but a more cautious approach is adopted to aldosterone antagonists. This reflects the high risk of hyperkalaemia observed in its referenced studies reaching up to 36% in certain population‐based registries and an associated increase in mortality 69. These findings are supported by the UK national heart failure audit, which lists serum potassium >5.5 mmol l^–1^ as the main predictor for mortality in inpatients with heart failure 2. This encourages a stricter schedule of electrolyte monitoring with aldosterone antagonist prescription, which is mirrored in this guideline.

## Heart failure medications, renal effects and monitoring according to guidelines

### Diuretics

Despite widely acknowledged potential detrimental effects of loop and thiazide diuretics on renal function, only the ESC guidelines provide specific monitoring advice regarding their use in heart failure. This contrasts with guidance regarding aldosterone antagonists, which is comprehensive and specific in each guideline, reflecting evidence for risk of hyperkalaemia, which is a predictor of mortality 70, 71. Furthermore, prolonged use of spironolactone in patients with heart failure can increase risk of developing CKD, leading to this potassium‐sensitive state much sooner 7. Stringent monitoring systems are therefore justified for this drug class.

With loop diuretics, the risks may actually be at least as great as with aldosterone antagonists. In patients with heart failure, use of loop diuretics is associated with more severe renal decline, higher risk of hospital admission and increased mortality rate. The renal decline is dose‐dependent with higher doses causing more rapid decline in eGFR 7, 72. This association is increased in patients with WRF, causing even greater risk of mortality and dose‐dependent renal decline 73. It is not surprising then that increased use and requirement for both loop and thiazide diuretics are associated with increased risk of end‐stage renal disease 7, 74.

Paucity of renal function monitoring guidance for loop and thiazide diuretics, compared with aldosterone antagonists, probably relates to the fact that trials of the latter were conducted relatively recently. Lack of guidance for renal monitoring with diuretics may partially explain why diuretics are the second most common cause for UK hospital admissions due to adverse drug reactions 6. If patients at highest risk of renal decline are given the highest doses, without specific monitoring advice, poor outcomes are not surprising. These risks should be emphasized as much for diuretics as for aldosterone antagonists, in current guidelines.

Pharmacokinetic studies show that different diuretic agents have different durations of action in patients with heart failure, with different times from initiation to observed pharmacological effect (Table 4). Studies looking at the timed response to the loop diuretic bumetanide have shown that its diuretic effect occurs <1 h after oral administration. Maximal effect is achieved after the first dose, with diminishing effect of subsequent oral doses (up to 25% less than the first dose for the same concentration) 75. These findings are supported by observed actions of the loop diuretic furosemide, which shows a maximal effect within 1.5 h of the first oral dose and reduced effect with the same dose given repeatedly 76. A similar effect has been seen with the thiazide diuretic hydrochlorothiazide 77. The greatest diuretic effect is seen with the first few doses, causing significant electrolyte shifts within the first 3 days of administration. This can lead to hypokalaemia, hyponatraemia and compensatory mechanisms for sodium retention, including aldosterone release, thus effectively counteracting the diuretic effect. A new steady state is achieved after about 2 weeks where salt intake and natriuresis are balanced.

**Table 4:** Duration of action of diuretic agents used in heart failure 68

Loop diuretics	Maximum daily dose	Duration of action
**Bumetanide**	10 mg	4–6 h
**Furosemide**	600 mg	6–8 h
**Torsemide**	200 mg	12–16 h

Thiazide diuretics	Maximum daily dose	Duration of action
**Chlorothiazide**	1000 mg	6–12 h
**Chlorthalidone**	100 mg	24–72 h
**Hydrochlorothiazide**	200 mg	6–12 h
**Indapamide**	5 mg	36 h
**Metolazone**	20 mg	12–24 h

These observations have implications for both diuretic dosing and monitoring of renal function. The greatest change in renal function biomarkers (such as serum creatinine) would be expected after the first dose and higher subsequent doses of the drug may be required for the same effect. In a patient with symptomatic heart failure, this may result in a progressive increase in diuretic dose with an accompanying greater risk of decline in renal function and AKI. This is compounded by the need for increasing doses of thiazide and loop diuretics as GFR falls 57, 76. With reduced kidney perfusion, there is reduced rate of excretion of diuretic into the renal tubules, which is required for these drugs to reach their sites of action. In addition, progressive nephron loss in CKD results in fewer sites at which the diuretics can act 22. This not only reduces their effect as diuretics but also increases half‐life in CKD, which can cause *resistance* to the diuretic so that increasing doses are required over time 78. In addition, bioavailability of oral diuretics may be reduced in patients with heart failure because of the presence of gut wall oedema 76. Thus, patients with CKD and symptomatic fluid overload are faced with the highest initial risk of renal deterioration, which is further increased by their need for higher doses of diuretics. Often this leads to hospital admission for administration of IV diuretics.

No simple monitoring schedule is applicable for any one class of diuretic. Patient and drug factors are both important, with consideration of both the acute and maintenance phases of the diuretic action. A regimen of 1–2 weeks' monitoring (recommended by ESC) may reach the steady state effect of the diuretic, but it does not take account of the risk of chronic slow deterioration in renal function. As discussed, monitoring of adequate frequency and duration is not reflected in current guidelines from NICE, SIGN and AHA, which rely predominantly on clinician judgement.

### ACEis, ARBs and antihypertensives

The effects of ACEis and ARBs on renal function are well documented 52 and current heart failure guidelines emphasize the importance of renal function monitoring, suggesting no more than 2 weeks between initiation and first follow‐up test. Slow titration is recommended at 2‐week intervals with close follow‐up after dose changes. This strategy facilitates detection of a sudden fall in renal function after drug initiation or a progressive deterioration with dose increments. Higher doses of ACEis are associated with risk of greater acute rises in creatinine over the first 4 months of therapy, so relatively frequent monitoring is justified over this period 79. Some studies advocate monitoring within 3–7 days of initiation of ACEis, to capture the first dose effect. This may be particularly relevant in patients with CKD (or transiently impaired renal function) and high baseline serum potassium, where there is relatively high risk of hyperkalaemia, which can increase risk of mortality 80. For this reason, the guidelines do advise caution for such patients. While the monitoring advice during the titration period is appropriate, the risks of first dose effects could be better observed with earlier follow up after initiation such as post‐first dose monitoring.

For β‐blockers and other antihypertensives that may be used in heart failure, only SIGN provides specific advice on renal monitoring. Other guidelines discuss symptomatic side‐effects of β blockade such as dizziness, fatigue and hypotension but do not advise specifically on monitoring of renal function. This demonstrates one of the problems of a predominantly *medication‐based* prescribing (and monitoring) approach in a complex condition such as heart failure.

## Medication‐based *vs.* patient‐based monitoring

The approach of current heart failure guidelines to renal function monitoring can be described as *medication based*. With scarcity of evidence, much of the guidance reflects expert opinion or adverse effect data from clinical trials. No studies have specifically addressed optimal timing of renal function monitoring, which has contributed to the potentially confusing variation in the published medication‐based guidance.

For example, if a patient is taking a stable dose of spironolactone and then starts an ACEi, causing serum potassium to increase to 5.6 mmol l^–1^, SIGN, ESC and ACCF would recommend halving of the spironolactone dose rather than review of the ACEi, even though the temporal relationship might suggest that the ACEi was the main adverse factor for this individual patient.

Linking monitoring guidance to individual drug classes disassociates it from the patient, so that if one particular drug is discontinued, the guidance is assumed to stop with it. This creates the danger that the role of the heart failure pathology itself in causing renal deterioration will be ignored. Monitoring may become more concerned with detection of adverse effects rather than detection and reduction of progressive decline in renal function. This is reflected in current guidance for patients with apparently stable renal function, where only NICE has specific advice (of 6‐monthly testing). This does not take account of individual patient risk factors such as age and comorbidities.

In addition, guideline development is complicated by the fact that most patients with heart failure are prescribed multiple drugs (ACEis, spironolactone, loop diuretics, β‐blocker etc.). Few studies have addressed the effect of such complex combinations on renal function in the medium–long term. For those studies that have, exclusion criteria might render the conclusions inapplicable to many patients with comorbidities.

To rationalize guidance for monitoring of renal function and to minimize risk of WRF and AKI in vulnerable patients, a *patient‐based* monitoring regimen should be developed. This would consider both medication and individual risk factors, and suggest a monitoring interval based on a patient's combined risk, facilitating early intervention (such as dose adjustment) to reduce risk of renal deterioration, hospital admission and mortality.

## Limitations of monitoring renal function

Serum creatinine has, for decades, remained by far the most widely used assay for measuring renal function in primary and secondary care. It is a product of normal striated muscle turnover and is a marker of renal filtration function rather than of acute renal damage, because to a large extent it is filtered freely by the glomerulus. Despite the longevity of this assay, creatinine is far from ideal as a marker of renal function. Firstly, it does not increase linearly with fall in GFR; indeed GFR can fall significantly below normal with little or no increase in serum creatinine. Secondly, it is also inaccurate at low GFR because the small degree of active tubular secretion of creatinine can confound the serum level. Conversely, drugs such as trimethoprim can cause spuriously high serum creatinine by blocking its tubular secretion. Thirdly, creatinine is a particularly poor marker of renal function at extremes of muscle mass. Serum creatinine of 130 μmol l^–1^ might represent normal GFR in a young person with high muscle mass or very low GFR in an older malnourished person.

It is also essential to understand that while increase in serum creatinine over time suggests deterioration in renal function (and defines AKI and its stages), it does not provide any information on the underlying renal pathology. To take contrasting examples, a 50% increase in creatinine from baseline might result from dehydration, with no histological changes, or it might result (albeit rarely) from an intrinsic renal pathology such as glomerulonephritis. Clearly the clinician needs to apply clinical context and common sense to interpretation of the creatinine result. Identification of more specific blood and urine biomarkers for renal injury and function is currently highly topical and of commercial interest but none is likely to be used widely in the next few years.

Estimated GFR is derived from serum creatinine using formulae that include age, sex and ethnicity. Different formulae are described, the most commonly used being MDRD and CKD‐EPI. A key point about use of eGFR is that it was developed and validated in populations with steady or slowly declining renal function. It is valid to use eGFR to monitor renal function over months and years, but for more acute changes in renal function, serum creatinine should be used.

In monitoring renal function in the context of initiation and titration of drugs, often the trend in creatinine (or eGFR over months) is more important than the absolute value. Serum creatinine rising from 100 μmol l^–1^ to 200 μmol l^–1^ over 6 months following introduction and titration of a drug is likely to be of greater concern than serum creatinine that has remained stable at 220 μmol l^–1^ over the same period.

### Clinical factors which impact WRF

Personal risk factors other than medication need to be considered for effective patient‐based monitoring. These include sex, age, smoking status and comorbidities such as diabetes and CKD 24. By combining demographic factors such as sex and ethnicity with clinical history and comorbidities, risk can be assessed more comprehensively than by considering only the drug regimen. Decision rules have not been developed for patients in the community with heart failure presumably because of the complexity of dealing with multiple risk factors. The lack of data from sufficiently large cohorts has also been a limiting factor, but may be easier with increasing adoption of electronic patient records.

### Rate of renal decline

Rate of renal decline is itself a risk factor for further renal deterioration. Serum creatinine results inevitably fluctuate in patients with heart failure, often because of changes in their cardiac condition and associated changes in their drug regimen. Comorbidities and other acute illnesses may also contribute to variation of creatinine between time points. However, the underlying or *baseline* renal function nearly always declines over years, as is the natural history of most aetiologies of CKD. The average annual fall in eGFR over 4 years can be used as a measure of the rate of long‐term decline 59, and can act as a prognostic indicator for both the heart failure and the renal outcome. For example, a rapid decline in baseline renal function is associated with increased risk of incident heart failure, before the diagnosis of heart disease 81, 82. Furthermore, patients with higher rate of renal decline have higher risk of mortality associated with heart failure compared with those with a slower renal decline 83, 84. There might therefore be some benefit in standardization of this rate of decline, to facilitate risk stratification.

### Timing of intervention (intervention lag)

This is the time taken from initiation of monitoring of renal function to a change in clinical management to avoid an undesirable outcome. Typically, this would simply involve the time taken for a patient to come back to clinic for a blood test, the waiting time for the result and the subsequent action which might involve another clinic visit or telephone call. In primary care this can be time consuming, especially if it relies on transportation of blood samples to a hospital laboratory. With further time for patient recall, the intervention lag could range from 2–5 days. The delay could be reduced with telephone follow‐up and use of recent advances in telemonitoring of clinical symptoms and signs 85. Taking this intervention lag into account is essential in planning a monitoring schedule.

## Personalized monitoring guidance – a new perspective

By incorporating a patient's drug regimen, personal risk factors, baseline renal function and time required to intervene, a flexible monitoring system could be developed to improve renal outcomes in patients at high risk. Simply increasing frequency of renal monitoring for all patients with heart failure would have significant cost implications and is unrealistic in a publicly funded health service. Identification of patients at greatest risk of renal decline together with more efficient monitoring systems is likely to be the most cost‐effective way of improving outcomes including hospital admission.

This type of personalized care requires an automated system to analyse individual risk factors and derive clinical guidance for each patient. This could be aided by recent advances in machine learning and virtual‐intelligence‐aided clinical‐decision tools, which allow both rapid and intelligent assessment of biomarkers for clinical use 86. For example, predictive analytics may be able to identify trends in eGFR and predict onset of WRF, calculating optimal monitoring time intervals and advising on dose adjustments to protect renal function. This personalized monitoring would take into account both early effects of medication changes and chronic effects of the disease process. Since it would be based on the individual patient, it would use cumulative data to improve its predictive accuracy with time 87. Advances in remote care will help by bridging the gap between patient and clinician. This concept can be illustrated by the current work being developed by Google Deepmind's *Stream* application, which collates hospital data from numerous sources, and uses predictive analytics to identify patients at highest risk of deterioration, allowing early intervention to avoid AKI in hospital inpatients 88. Allowing potential real‐time monitoring of renal function and instant medical feedback in this way would result in safer drug prescribing and lower risk of renal complications 89.

## Conclusions

Renal dysfunction contributes substantially to morbidity and mortality in patients with heart failure 90. Current national and international CHF guidelines on renal function monitoring focus on drugs and their potential adverse effects, but offer less specific advice about monitoring of renal function. This reflects a paucity of data from adequately powered clinical studies. Risk factors for deterioration of renal function in CHF have been identified and could be incorporated into a *patient‐based* approach to monitoring.

## Competing Interests

The views expressed are those of the authors and not necessarily those of the NHS, the NIHR or the Department of Health. M.P. is an NIHR Senior Investigator and is also supported by the MRC Centre for Drug Safety Science.

*This research was supported by the National Institute of Health Research Collaboration for Leadership in Applied Health Research and Care North West Coast (NIHR CLAHRC NWC).*

## Supporting information


**Appendix S1** Search strategies for databases used in this review
