# Supplementary material for: Renal function monitoring in heart failure – what is the optimal frequency? A narrative review
Source: Br J Clin Pharmacol. 2017 Oct 22;84(1):5–17. doi: 10.1111/bcp.13434 (PMC5736847; doi:10.1111/bcp.13434)
Supplement: Supplementary file 1 — Appendix S1 Search strategies for databases used in this review [file BCP-84-5-s001.docx]

| **Appendix A – Search strategies for ‘Renal function monitoring in heart failure – what is the optimal frequency? A narrative review’** |
| --- |

| **Database: CDSR/Central/ DARE/HTA** |
| --- |
| #1 MeSH descriptor: [Heart Failure] explode all trees 6571  #2 MeSH descriptor: [Edema, Cardiac] explode all trees 22  #3 MeSH descriptor: [Heart Failure, Diastolic] explode all trees 31  #4 MeSH descriptor: [Heart Failure, Systolic] explode all trees 143  #5 ((heart* or cardiac* or myocardial* or ventric*) near/2 (fail* or decompensat* or insuffic* or dysfunc*)) 20023  #6 #1 or #2 or #3 or #4 or #5 20032  #7 MeSH descriptor: [Glomerular Filtration Rate] explode all trees 2203  #8 (glomerul* filtrat* rate*) 5129  #9 #7 or #8 5129  #10 #6 and #9 605  #11 time or frequenc* 260059  #12 #10 and #11 234 |

| **Database: Medline** |
| --- |
| Strategy used:   \| 1 \| heart failure/ or edema, cardiac/ or heart failure, diastolic/ or heart failure, systolic/ \| 99711 \| \| --- \| --- \| --- \| \| 2 \| ((heart* or cardiac* or myocardial* or ventric*) adj2 (fail* or decompensat* or insuffic* or dysfunc*)).tw. \| 175303 \| \| 3 \| 1 or 2 \| 201910 \| \| 4 \| glomerular filtration rate/ \| 37180 \| \| 5 \| (glomerul* adj1 filtrat* adj1 rate*).tw. \| 34172 \| \| 6 \| 4 or 5 \| 52142 \| \| 7 \| 3 and 6 \| 2543 \| \| 8 \| animals/ not humans/ \| 4271460 \| \| 9 \| 7 not 8 \| 2378 \| \| 10 \| (time or frequenc*).tw. \| 3071275 \| \| 11 \| 9 and 10 \| 339 \| |

| **Database: Embase** |
| --- |
| Strategy used:   \| 1 \| heart failure/ or edema, cardiac/ or heart failure, diastolic/ or heart failure, systolic/ \| 178169 \| \| --- \| --- \| --- \| \| 2 \| ((heart* or cardiac* or myocardial* or ventric*) adj2 (fail* or decompensat* or insuffic* or dysfunc*)).tw. \| 256795 \| \| 3 \| 1 or 2 \| 304803 \| \| 4 \| glomerular filtration rate/ \| 44915 \| \| 5 \| (glomerul* adj1 filtrat* adj1 rate*).tw. \| 43628 \| \| 6 \| 4 or 5 \| 63953 \| \| 7 \| 3 and 6 \| 4753 \| \| 8 \| animals/ not humans/ \| 1172721 \| \| 9 \| 7 not 8 \| 4734 \| \| 10 \| (time or frequenc*).tw. \| 3740110 \| \| 11 \| 9 and 10 \| 748 \| |

| **Database: Web of science** |
| --- |
| Strategy used:  **TITLE:** ((heart* or cardiac* or myocardial* or ventric*))*AND* **TITLE:** ((fail* or decompensat* or insuffic* or dysfunc*)) *AND* **TOPIC:**(glomerul* filtrat* rate*) *AND* **TOPIC:**(time or frequenc*) |
| Database: Web of science |
| Strategy used:   \| 1 \| CINAHL \| exp HEART FAILURE/ \| [19918](http://www.library.nhs.uk/hdas/search-results/1?PageNumber=1&PageSize=10&SortBy=srt.unspecified&ShowAbstracts=False&BackToSearchResultsCount=1&databases=bnj.ebs.cinahl) \| \| --- \| --- \| --- \| --- \| \| 2 \| CINAHL \| (((heart* OR cardiac* OR myocardial* OR ventric*) adj2 (fail* OR decompensat* OR insuffic* OR dysfunc*))).ti,ab \| [24727](http://www.library.nhs.uk/hdas/search-results/2?PageNumber=1&PageSize=10&SortBy=srt.unspecified&ShowAbstracts=False&BackToSearchResultsCount=1&databases=bnj.ebs.cinahl) \| \| 3 \| CINAHL \| 1 OR 2 \| [30876](http://www.library.nhs.uk/hdas/search-results/3?PageNumber=1&PageSize=10&SortBy=srt.unspecified&ShowAbstracts=False&BackToSearchResultsCount=1&databases=bnj.ebs.cinahl) \| \| 4 \| CINAHL \| exp GLOMERULAR FILTRATION RATE/ \| [3584](http://www.library.nhs.uk/hdas/search-results/4?PageNumber=1&PageSize=10&SortBy=srt.unspecified&ShowAbstracts=False&BackToSearchResultsCount=1&databases=bnj.ebs.cinahl) \| \| 5 \| CINAHL \| ((glomerul* adj1 filtrat* adj1 rate*)).ti,ab \| [2894](http://www.library.nhs.uk/hdas/search-results/5?PageNumber=1&PageSize=10&SortBy=srt.unspecified&ShowAbstracts=False&BackToSearchResultsCount=1&databases=bnj.ebs.cinahl) \| \| 6 \| CINAHL \| 4 OR 5 \| [4861](http://www.library.nhs.uk/hdas/search-results/6?PageNumber=1&PageSize=10&SortBy=srt.unspecified&ShowAbstracts=False&BackToSearchResultsCount=1&databases=bnj.ebs.cinahl) \| \| 7 \| CINAHL \| 3 AND 6 \| [400](http://www.library.nhs.uk/hdas/search-results/7?PageNumber=1&PageSize=10&SortBy=srt.unspecified&ShowAbstracts=False&BackToSearchResultsCount=1&databases=bnj.ebs.cinahl) \| \| 8 \| CINAHL \| (time OR frequenc*).ti,ab \| [273088](http://www.library.nhs.uk/hdas/search-results/8?PageNumber=1&PageSize=10&SortBy=srt.unspecified&ShowAbstracts=False&BackToSearchResultsCount=1&databases=bnj.ebs.cinahl) \| \| 9 \| CINAHL \| 7 AND 8 \| [49](http://www.library.nhs.uk/hdas/search-results/9?PageNumber=1&PageSize=10&SortBy=srt.unspecified&ShowAbstracts=False&BackToSearchResultsCount=1&databases=bnj.ebs.cinahl) \| |
